# Different responses of abundant and rare bacterial composition to groundwater depth and reduced nitrogen application in summer maize field

**DOI:** 10.3389/fmicb.2023.1220731

**Published:** 2023-10-13

**Authors:** Fangfang Bai, Wei Guo, Ping Li, Dongmei Qiao, Zhenjie Du, Xuebin Qi

**Affiliations:** ^1^Institute of Farmland Irrigation, Chinese Academy of Agricultural Sciences, Xinxiang, China; ^2^Water Environment Factor Risk Assessment Laboratory of Agricultural Products Quality and Safety, Ministry of Agriculture and Rural Affairs, Xinxiang, China; ^3^Agricultural Water Soil Environmental Field Research Station of Xinxiang, Chinese Academy of Agricultural Sciences, Xinxiang, China

**Keywords:** bacterial composition, abundant and rare bacteria, groundwater depth, reduced nitrogen application, summer maize field

## Abstract

**Introduction:**

It is well known that reduced nitrogen application and groundwater depth can change soil microbial communities, but the associated difference in the response of abundant and rare bacterial composition to these local environmental changes remains unclear.

**Methods:**

In this study a lysimeter experiment was carried out to examine the impact of reduced nitrogen and groundwater depth on the composition of abundant and rare bacteria.

**Results and discussion:**

Our results demonstrated that the summer maize field soil species composition of rare bacterial sub-communities was significantly regulated by reduced nitrogen application, groundwater depth change and their interactions. However, only reduced nitrogen application had a significant influence on the species composition of abundant bacterial sub-communities. The structural equation model (SEM) indicated that reduced nitrogen application and groundwater depth change also could indirectly regulate the species composition of abundant and rare bacteria by altering soil attributes. The changes in soil pH and TSN had the most significant effects on the community composition of abundant and rare bacteria, respectively. More importantly, rare bacterial sub-communities were more sensitive to the changes in nitrogen input, groundwater depth and soil factors. Collectively, our study first demonstrated that abundant and rare microbial sub-communities responded differently to reduced nitrogen application and groundwater depth change. This study highlights that summer maize farmland production management should take nitrogen input and groundwater depth into consideration to maintain the compositional stability of soil rare microbial sub-communities.

## 1. Introduction

Soil microorganisms play a major role in regulating key ecosystem processes and functions (Koranda et al., 2013; Mooshammer et al., 2014). Nitrogen is one of the most important limiting factors for the growth of soil microbes (Sun et al., 2015; Hou et al., 2018; Zhang et al., 2022). Nitrogen fertilizers can not only directly affect soil microbial communities but also indirectly affect soil microbial communities by regulating soil fertility, soil salt content and groundwater salt content (She et al., 2018; Bai et al., 2020; Wu et al., 2020; Bai et al., 2023). However, over-application of nitrogen fertilizers may result in a series of severe environmental problems, including soil salinization, nitrate contamination and groundwater pollution (Zhang et al., 2013; Lasagna et al., 2016). In addition, over-application of nitrogen fertilizers may greatly negatively affect the function of ecosystems by altering soil bacterial diversity and composition (Luo et al., 2016; Cocco et al., 2018; Vergine et al., 2023). Therefore, it is necessary to explore the appropriate nitrogen input amount to maintain sustainable agricultural development (Ju et al., 2009; Liu B.-Y. et al., 2021).

Soil bacterial communities are primarily dominated by a few abundant species, whereas a great number of other species (“rare biosphere”) have extremely low abundance (Pedrós-Alió, 2012; Ji et al., 2020; Wang et al., 2021b). It is widely believed that abundant subcommunities mostly determine ecosystem functioning (Egidi et al., 2019). Most rare communities can be widely present in the microbial communities of various habitats, and they play an important role in regulating ecosystem stability and function (Liu et al., 2015; Jiao et al., 2017). Rare subcommunities account for only the entire microbial community, However, their contribution to the temporal dynamic change of community structure can reach 97% (Shade et al., 2014). The spatial distributions and driving factors of abundant and rare bacteria have been well examined in different ecosystem types, such as agricultural land (Wan et al., 2020; Jiao and Lu, 2020b), forest (Wang et al., 2021a), alpine grassland (Ji et al., 2020), wetland (Wan et al., 2021), and desert (Wang et al., 2021b). Abundant and rare bacterial species may be subjected to different determinants, and thus exhibit distinct ecological patterns (Wan et al., 2020; Jiao and Lu, 2020b; Wang et al., 2021b). Hence, comparing the response of abundant and rare bacterial sub-communities to reduced nitrogen application may help us better predict how soil biodiversity and function respond to global change. However, how differently reduced nitrogen application affects the species composition of abundant and rare bacteria in agricultural soils remains largely unclear.

As one of the most important crop-producing regions of China, the North China Plain has a total area of 300,000 km^2^. In the past decades, its groundwater consumption increased sharply because of population growth, urbanization and development of industry and agriculture (Changming et al., 2001; Chen et al., 2020; Jing et al., 2023; Xiao et al., 2023), causing various environmental geologic problems, including groundwater table fall, water quality worsening and surface subsidence (Changming et al., 2001; Shi et al., 2020; Gan et al., 2022). Notably, groundwater table can regulate soil microbial species richness and composition (Bai et al., 2020; Wu et al., 2020; Zhang et al., 2020). Besides, groundwater depth change can indirectly influence soil microbial communities by altering soil attributes such as salinity, pH and nutrients (Bai et al., 2020; Zhang et al., 2020). The interactions between reduced nitrogen application and groundwater depth have a significant influence on soil microbial communities (Bai et al., 2020). Furthermore, over-nitrogen form release may lead to severe groundwater contamination (Matzeu et al., 2017; Paladino et al., 2020; Abbasi and Sepaskhah, 2023). However, to date, the effect of reduced nitrogen application, groundwater depth and their interactions on the species composition of abundant and rare bacteria has been rarely studied.

Soil microbial communities may be determined by a wide range of soil attributes such as soil pH, salinity and nutrients (Bai et al., 2020; Wan et al., 2020; Jiao and Lu, 2020b; Wang et al., 2021b). In addition, the effects of different soil factors vary significantly between abundant and rare sub-communities (Jiao and Lu, 2020b; Wang et al., 2021b). For example, the assemblies of abundant and rare sub-communities are regulated by soil pH and soil moisture content, respectively (Wang et al., 2021a). Moreover, nitrogen application amount and groundwater depth and their interactions can cause substantial changes in soil attributes (Bai et al., 2020). We therefore posited that abundant and rare bacteria had different responses to the alteration in soil attributes caused by reduced nitrogen application and groundwater depth. However, few studies have focused on the direct and indirect effect of reduced nitrogen application, groundwater depth and soil attributes on abundant and rare bacteria in agricultural soils.

Therefore, the major objective of our study was to examine the associated difference in the response of abundant and rare bacteria to reduced nitrogen application and groundwater depth in agricultural soils. In this study, a large lysimeter experiment was conducted. Soil abundant and rare bacterial sub-communities were assessed based on 16S rRNA gene sequence data. Specifically, this study was designed to address the following questions: (1) How does the species composition of abundant and rare bacteria respond to reduced nitrogen application, groundwater depth and their interactions? (2) What are the direct and indirect influences of reduced nitrogen application, groundwater depth change and soil factors on soil abundant and rare bacterial composition?

## 2. Materials and methods

### 2.1. Experimental site and design

**Figure 1:**
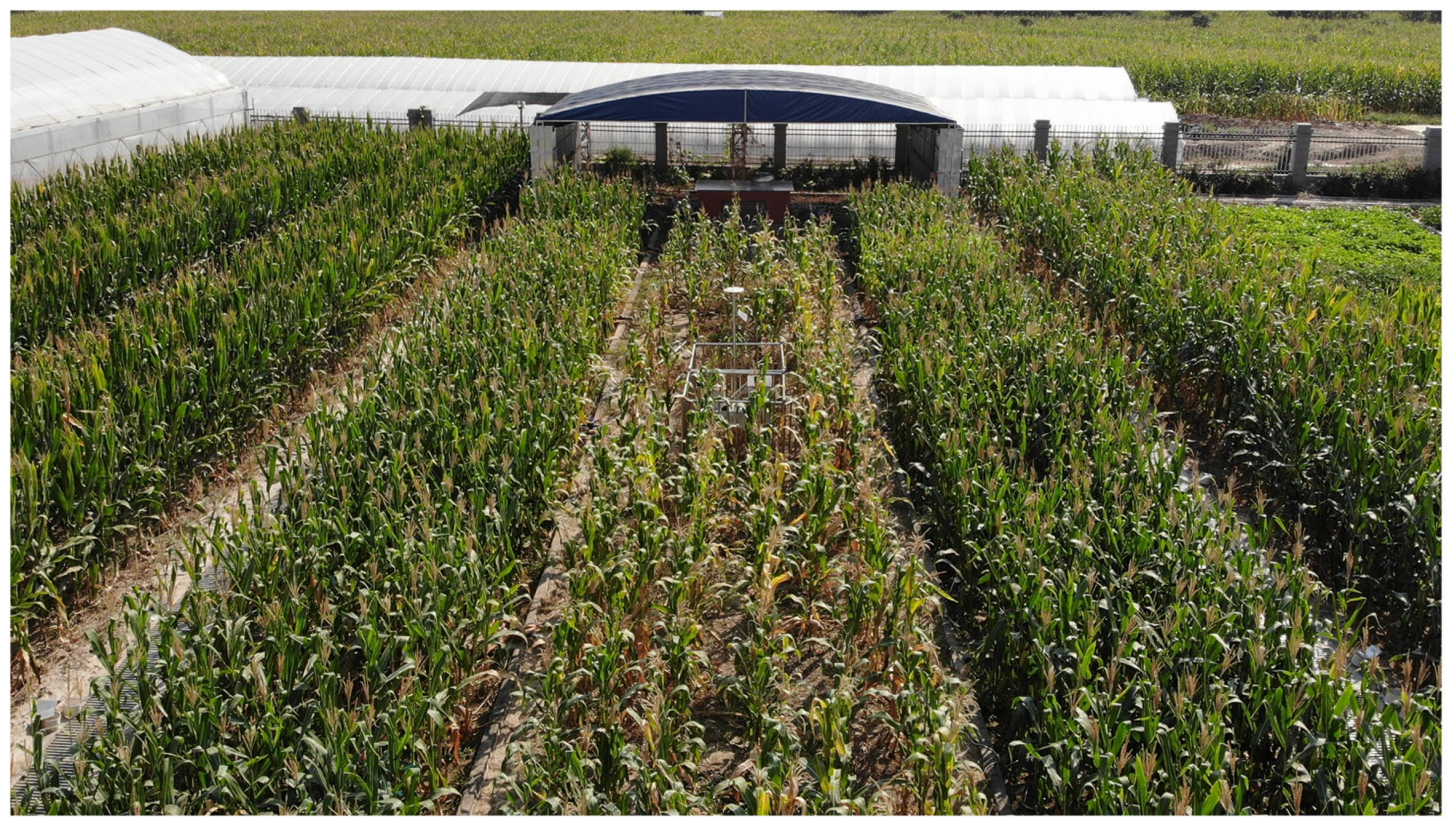
Above ground part of the lysimeter.

The experiment was carried out in the long-term monitoring lysimeter at the Xinxiang Agricultural Water and Soil Environment Field Scientific Observation and Experiment Station, Chinese Academy of Agricultural Sciences (35°19′N, 113°53′E), Xinxiang City, Henan Province, People’s Republic of China (Figure 1). The planting mode was winter wheat-summer maize two-cropping rotation a year. Its mean annual air temperature and annual precipitation is 14.1°C, 588.8 mm, respectively. Almost all precipitation is concentrated in July, August and September. The mean annual evapotranspiration is approximately 2,000 mm. The experiments were conducted on a large lysimeter platform in 2019, and each lysimeter was evenly filled layer by layer with silt loam soil collected from the neighboring cropland. The groundwater level in the micro-lysimeters was controlled at the constant depths of 2, 3, and 4 m from the soil surface by Mariotte flash.

The field site was sown with maize (*Zea mays* L.), the maize variety tested was Huaiyu 208, which was selected and bred by Henan Huaichuan Seed Industry Co., Ltd., and its approval number was Yu Shenyu 2,013,008, where we examined six treatments which were produced by the interactions between two levels of N input [local conventional N of 300 kg N ha^−1^ y^−1^ (N300), reduced N of 240 kg N ha^−1^ y^−1^ (N240)] and three groundwater depth levels (GW2, 2 m; GW3, 3 m and GW4, 4 m) (Table 1). Local conventional nitrogen level (300 kg N ha^−1^ y^−1^) was used as a control with 100% of normal nitrogen input. Maize was sown on June 10, 2020 and harvested on September 23, with a growth period of 105 days. Forty five millimeter water were irrigated immediately after sown. Excessively shallow groundwater depth will limit the growth of crops and root aerobic respiration, and lead to soil waterlogging (Askri et al., 2014; Soylu et al., 2014). A previous study has observed that 2.5 m is a crucial groundwater depth to control soil salinization (Askri et al., 2014). When the groundwater table is shallow, there are obvious two-ways of water exchange at the interface under the root layer, the upper soil moisture can infiltrate into deep soil and ground water, and crop evapotranspiration can partially utilize the groundwater. When the groundwater table becomes deeper, the soil moisture at the interface mainly migrates downward, and the critical groundwater depth for crop growth in the North China Plain is generally 4 m. Therefore, we used 2 m, 3 m and 4 m as the treatments of groundwater depth in our tests. The experiment followed a random block design with four replicates. Furthermore, 120 kg K_2_O ha^−1^ and 150 kg P_2_O_5_ ha^−1^ were applied in six treatments in the form of potassium sulfate and monocalcium phosphate, respectively. Nitrogen fertilizer was urea with 46% nitrogen content. Base fertilizer were applied on June 29 of 2020 (14, BBCH-scale), and topdressing were applied on July 29 of 2020 (51, BBCH-scale). The ratio of base fertilizer and topdressing of nitrogen was 4:6, phosphate fertilizer and potassium fertilizer were applied as base fertilizer at one time. Fertilization immediately followed by irrigation. And ground irrigation was conducted when the soil moisture was lower than 55% of the field water holding rate. A RS-XAJ-100 probe was buried in the 20 cm soil layer to monitor soil moisture and guide irrigation. The whole growth period of maize was irrigated 45 mm on June 10, June 29, July 15, July 29, August 10 and September 4, respectively.

**Table 1:** The experimental design.

Nitrogen application amount	Groundwater depth		
	2 m	3 m	4 m
240 kg·hm^−2^	GW2N240	GW3N240	GW4N240
300 kg·hm^−2^	GW2N300	GW3N300	GW4N300

### 2.2. Sampling and soil chemical properties determination

At the September 24 of 2020, five soil samples (0–20 cm depth) were randomly collected from each lysimeter using an auger and then mixed into a composite sample. A total of 24 composite samples were collected in this study. After being sieved through a 2 mm mesh to remove roots and stones, each composite sample was divided into two following parts: one portion was stored in thermally insulated boxes to measure soil attributes, and the other was used to extract DNA. Afterwards, soil pH (pH), organic carbon content (TOC), total nitrogen content (TSN) and total phosphor (TSP) were determined following the previous methodology (Wang et al., 2020). Soil available phosphorus (AP) was extracted from 0.5 M NaHCO_3_ and determined by a colorimetric method. Meanwhile, soil electrical conductivity (EC) was measured by soil electrical conductivity. Finally, soil NH_4_^+^-N(NHN) and NO_3_^−^-N(NON) were determined based on the previous methodology (Li et al., 2019).

### 2.3. Molecular and bioinformatics analysis

The bacterial DNA was extracted from 0.5 g of well-mixed fresh soil samples using E.Z.N.A. soil DNA kits (OMEGA, United States) following the manufacturer’s instructions. The V3-V4 hypervariable regions of the bacterial 16S rRNA gene were amplified using the universal primers 338F (5′-ACTCCTACGGGAGGCAGCAG-3′) and 806R (5′-GGACTACHVGGGTWTCTAAT-3′). These primers contained a set of 8-nucleotide barcodes sequence unique to each sample. The PCR program was as follows: 94°C for 5 min, 25 cycles at 94°C for 30 s, 55°C for 30 s and 72°C for 30 s with a final extension of 72°C for 10 min. Moreover, PCR amplicons were extracted from 2% agarose gels and purified using the AxyPrep DNA Gel Extraction Kit (Axygen Biosciences, Union City, CA, United States) based on the manufacturer’s instructions, and quantified using QuantiFluor™ -ST (Promega, United States). PCR made with distilled water without a soil sample DNA template were set as a negative control NTC, if the negative control has amplified bands, this batch of PCR will be redone. Purified amplicons were pooled in equimolar and paired-end sequenced (2 × 300) on an Illumina Miseq PE300 sequencing platform at Beijing Allwegene Tech, Ltd. (Beijing, China).

Bacterial sequences >200 bp and with an average quality score > 20 and no ambiguous base calls were processed within the QIIME package. These high-quality sequences were clustered into operational taxonomic units (OTUs) based on a 97% similarity threshold using uparse. The taxonomy of each 16S gene sequence was analy*z*ed by comparing it against sequences within the SILVA reference database (v.138). Subsequently, we removed the that contained less than 20 reads to avoid random effects on the identification of rare taxa (Jiao and Lu, 2020b; Wan et al., 2021). Based on minimum number of sample sequences, sequences were rarefied at 31,124 sequences from each sample to eliminate the effect of sequencing depths on the analyses. The rare and abundant OTUs were defined following previous study (Jiao and Lu, 2020b; Wan et al., 2021). OTUs with a relative abundance of >0.1% of the total abundance were defined as abundant bacteria, and rare bacteria were those with a relative abundance of <0.01% (Mo et al., 2018; Wang et al., 2021b; Ren et al., 2022).

### 2.4. Statistical analyses

Bray-Curtis community difference matrices of abundant and rare bacteria (the abundance data was Hellinger-transformed) and standardized environmental Euclidean distance matrix for each variable were used for evaluation. The difference distance of Bray-Curtis community was used to reflect the difference in species composition (β-diversity) among soil bacterial communities. Permutational analysis of variance (PERMANOVA) was used to examine the differences in species composition (β-diversity) of abundant and rare bacteria across nitrogen-treatment and groundwater-depth groups. Moreover, the difference in community composition was visualized through Nonmetric multidimensional scaling (NMDS). Levins’ niche breadth (B) index was used to compare the niche breadth of abundant and rare bacterial sub-communities (Levins, 1968). Particularly, the B-value of each bacterial OTU was estimated using the previous methods (Jiao and Lu, 2020a). Abundance-weighted mean B-values across all bacterial OTUs within the community were employed to quantify community B-values (Bcom). The higher the B-value of community, the stronger the metabolic flexibility (Pandit et al., 2009). Notably, the Levins’ niche breadth (B) index was calculated within the “spaa” R package.

Mantel test (10,000 permutations) was used to explore the influence of soil properties on soil bacterial richness. The structural equation model (SEM) was used to analyze the indirect and direct influences of groundwater depth, nitrogen application amount and soil factors on abundant and rare bacterial composition. In order to reduce the strong collinearity between variables, soil pH, TSP and SAP were removed because of the high correlation between these variables (i.e., Pearson’s *r* > 0.6). Prior to the analysis, we built a prior structural equation model (Supplementary Figure S1) according to relevant theories. The comparative fit index (CFI), goodness of fit index (GFI), low root square mean error of approximation (RMSEA) and χ^2^-test were used to fit SEM. Additionally, the “lavaan” program was utilized to perform SEM.

## 3. Results

### 3.1. Overall distribution patterns of abundant and rare sub-communities

After chimeric sequences were filtered and removed, a total of 746,976 high-quality sequences were clustered into 4,619 bacterial OTUs (Supplementary Table S2), among which only 203 (4.39%) OTUs with 36,2,101 sequences (50.52%) were identified as abundant bacteria, while a total of 3,096 (67.03%) OTUs with 86,975 sequences (12.13%) were identified as rare bacteria. Abundant sub-communities were primarily dominated by *Pseudomonadota* (29.17%), *Actinobacteriota* (25.46%), *Acidobacteriota* (19.55%), and *Chloroflexota* (11.18%), while rare sub-communities were primarily dominated by *Chloroflexota* (14.66%), *Pseudomonadota* (14.21%), and *Actinobacteriota* (10.48%) (Supplementary Table S3).

**Figure 2:**
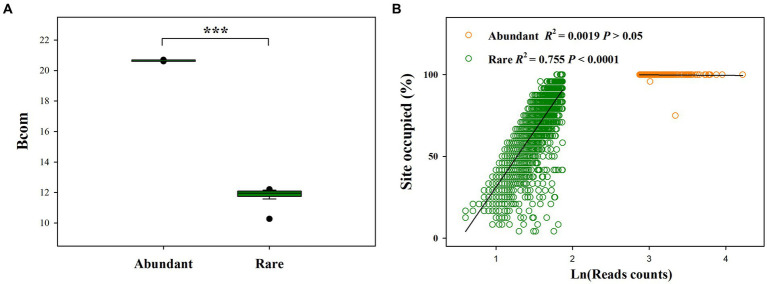
Difference in mean habitat niche breadths (Bcom, which was estimated to reveal the contributions of species sorting and dispersal limitation to microbial community assembly) between abundant and rare bacterial subcommunities **(A)**, and abundance-occupancy relationships for abundant and rare sub-communities **(B)**; ***p < 0.0001; Wilcoxon rank-sum test.

Our results showed that the mean Bcom value for rare sub-communities was significantly lower than that for abundant sub-communities (Figure 2A). Moreover, abundant species had a greater presence than rare species across six treatments. Particularly, 99.05% of the abundant OTUs (201 OTUs) occurred in all samples of six treatments, whereas only 0.32% of the rare OTUs (10 OTUs) occurred in all samples of six treatments (Figure 2B). In addition, abundance-occupancy relationships indicated that rare bacteria had a greater positive association than abundant bacteria.

### 3.2. Difference in the response of abundant and rare bacterial sub-communities to reduced nitrogen application and groundwater depth change

**Figure 3:**
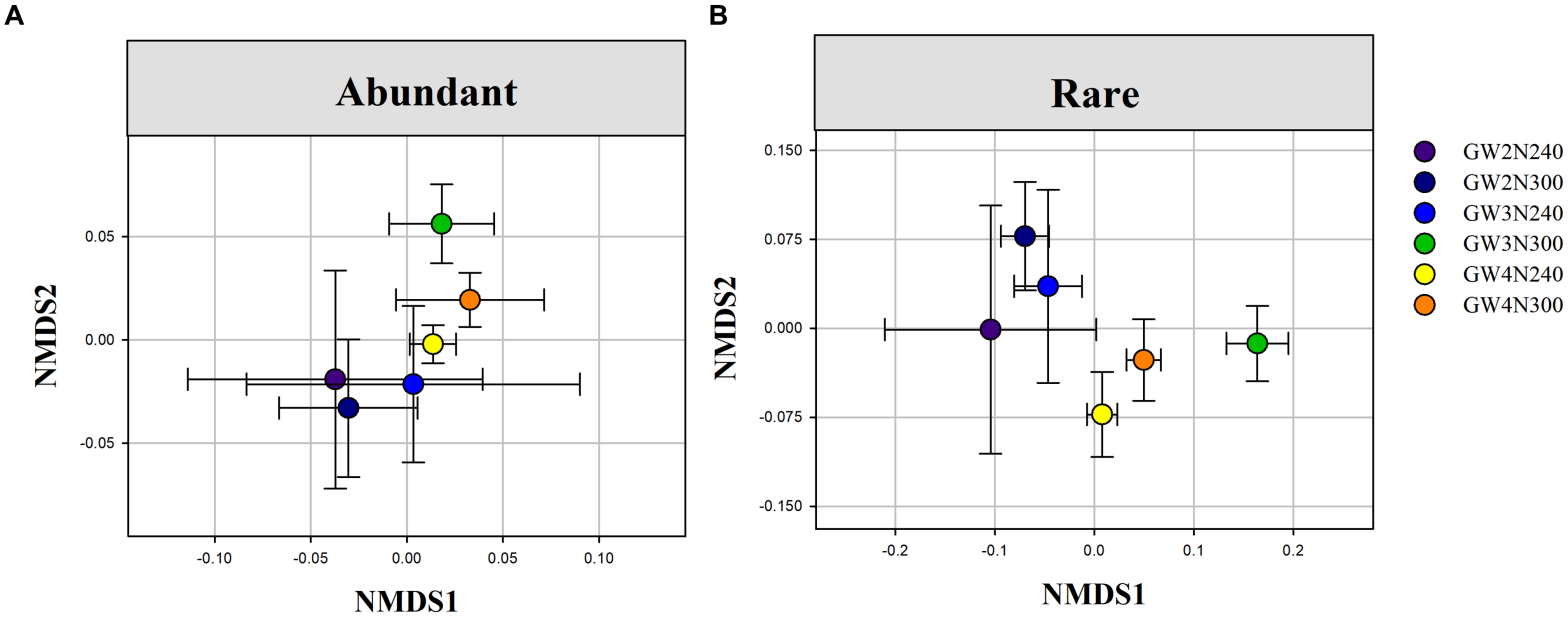
Nonmetric multidimensional scaling (NMDS) ordination of abundant **(A)** and rare **(B)** sub-community compositional differences (Bray-Curtis distance) under different groundwater depths and reduced nitrogen application. GW2N240, reduced N treatment × groundwater depth at 2 m; GW3N240, reduced N treatment × groundwater depth at 3 m;GW4N240, reduced N treatment × groundwater depth at 4 m; GW2N300, control with 100% of normal nitrogen input × groundwater depth at 2 m; GW3N300, control with 100% of normal nitrogen input × groundwater depth at 3 m; GW4N300, control with 100% of normal nitrogen input × groundwater depth at 4 m.

Results of PERMANOVA and NMDS showed that reduced nitrogen application significantly altered the species composition of rare sub-communities (*F* = 2.405, *p* < 0.0001), groundwater depth (*F* = 1.445, *p* < 0.01) and their interactions (*F* = 1.468, *p* < 0.01; Table 2 and Figure 3B). However, the species composition of abundant sub-communities only had a significant response to reduced nitrogen application (*F* = 3.724, *p* < 0.0001), but not to groundwater depth (*F* = 1.533, *p* > 0.05) or their interactions (*F* = 1.712, *p* > 0.05; Table 2 and Figure 3A). Abundant and rare sub-communities exhibited different shifts under N input and groundwater depth levels.

**Table 2:** Effects of groundwater depth, reduced nitrogen application and their interactions on the species composition of abundant and rare sub-communities.

Index		N input	GWD	Ninput*GWD
Abundant	*F*	3.724	1.533	1.712
	*P*	<0.0001	0.117	0.066
Rare	*F*	2.405	1.445	1.468
	*P*	<0.0001	<0.01	<0.01

N input, levels of N input; GWD, levels of groundwater depth; N input*GWD, interactions between N input and GWD; F, *F*-test value; P, probability value.

**Figure 4:**
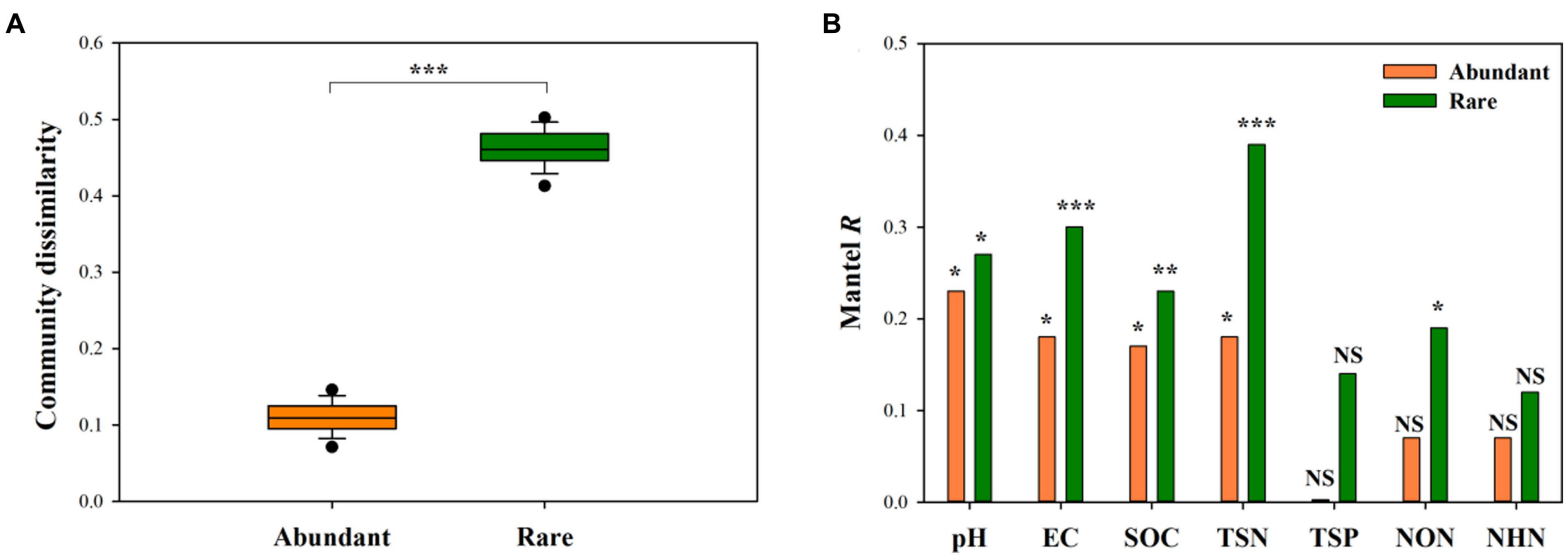
Difference in the compositional dissimilarity between abundant and rare sub-communities **(A)** and the response of the compositional dissimilarity of abundant and rare sub-communities to soil factors **(B)**. ***p < 0.001, **p < 0.01, *p < 0.05, ^NS^*p* > 0.05.

**Figure 5:**
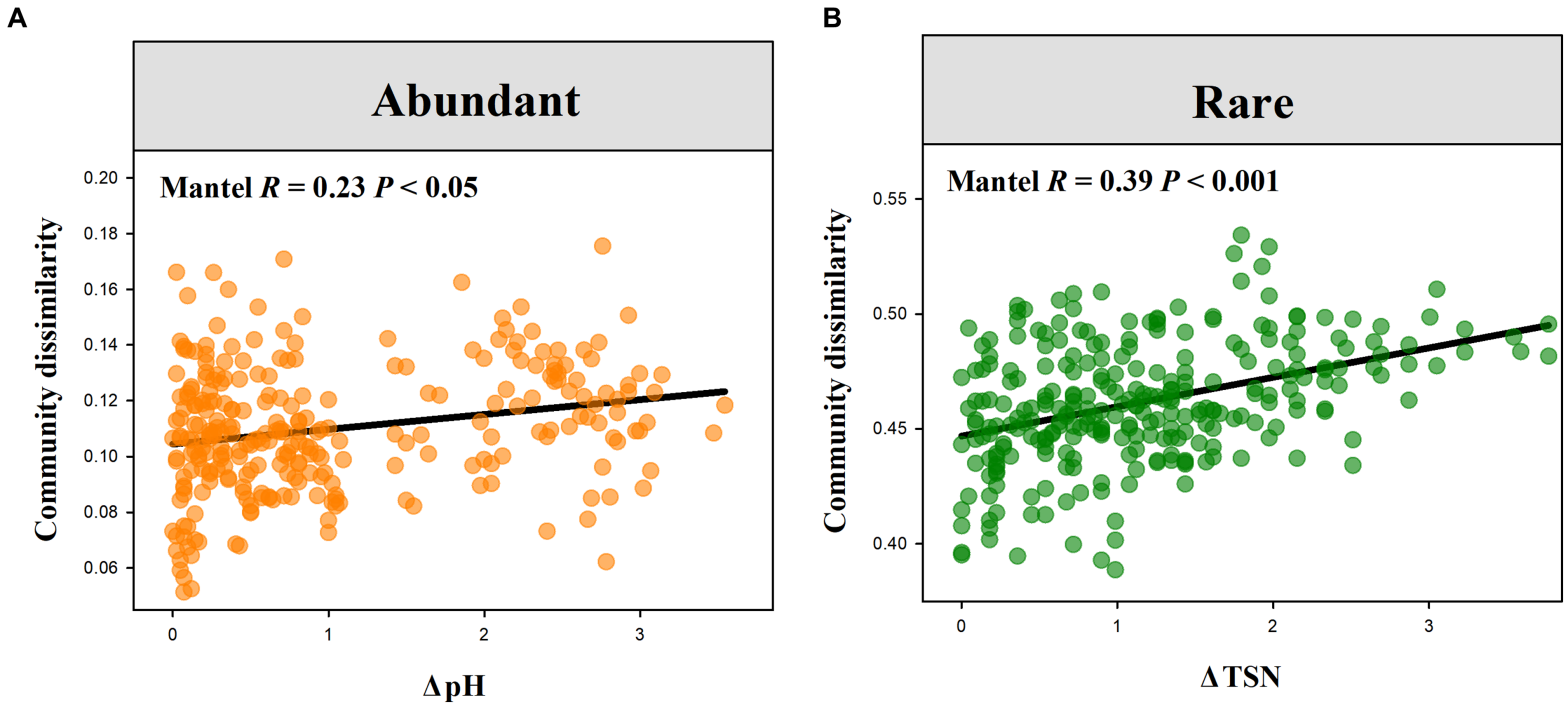
Relationships of soil abundant and rare bacterial compositional dissimilarity to the variations in soil pH **(A)** and TSN **(B)**, respectively.

Wilcoxon rank-sum test demonstrated that rare bacterial sub-communities had significantly greater compositional dissimilarity than abundant bacterial sub-communities (Figure 4A, *P* < 0.001). Mantel correlation analysis revealed that the species composition of abundant bacterial sub-communities had a significant response to the changes in soil pH, EC, SOC, and TSN (*p* < 0.05), whereas that of rare sub-communities exhibited a significant response to soil pH, EC, SOC, TSN, and NON (*p* < 0.05) (Figures 4B, 5). Moreover, we found that the strength of responses (i.e., Mantel *R*) in abundant and rare sub-community dissimilarity to the change in each soil variable was different (Figure 4B). Among all soil variables, soil pH and TSN played a major role in altering abundant and rare sub-community dissimilarity, respectively (Figures 4B, 5A,B).

### 3.3. Influence of reduced nitrogen application, groundwater depth change and soil attributes on soil abundant and rare bacterial composition

**Figure 6:**
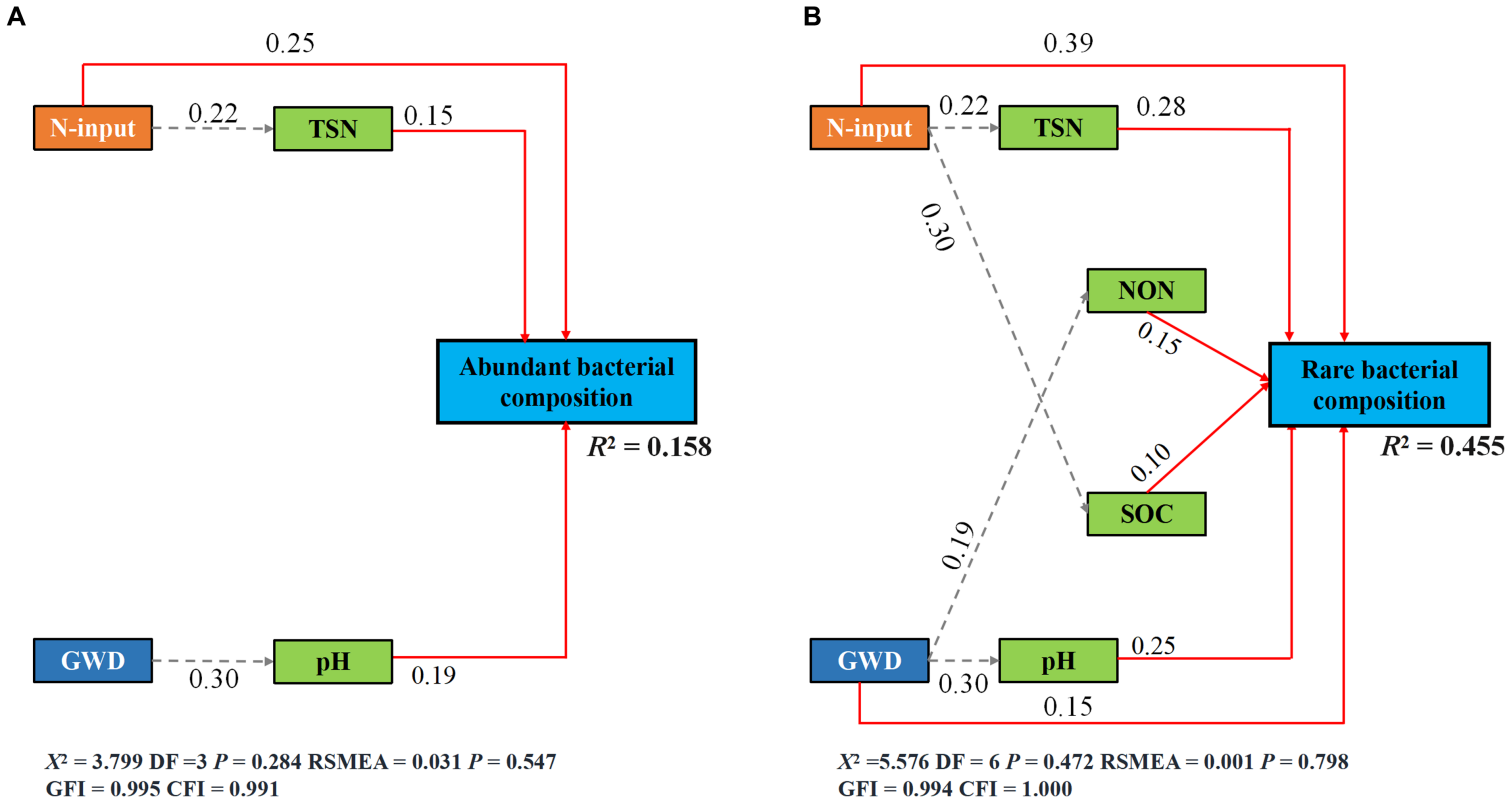
The species composition of abundant **(A)** and rare **(B)** subcommunities is described by SEM in terms of the direct and indirect effects of groundwater depth, reduced nitrogen application, and soil properties (Brey-Curtis community differences). Solid red and dashed gray arrows indicate the significant direct paths (*p* < 0.05) and indirect paths (*p* < 0.05), respectively.

**Figure 7:**
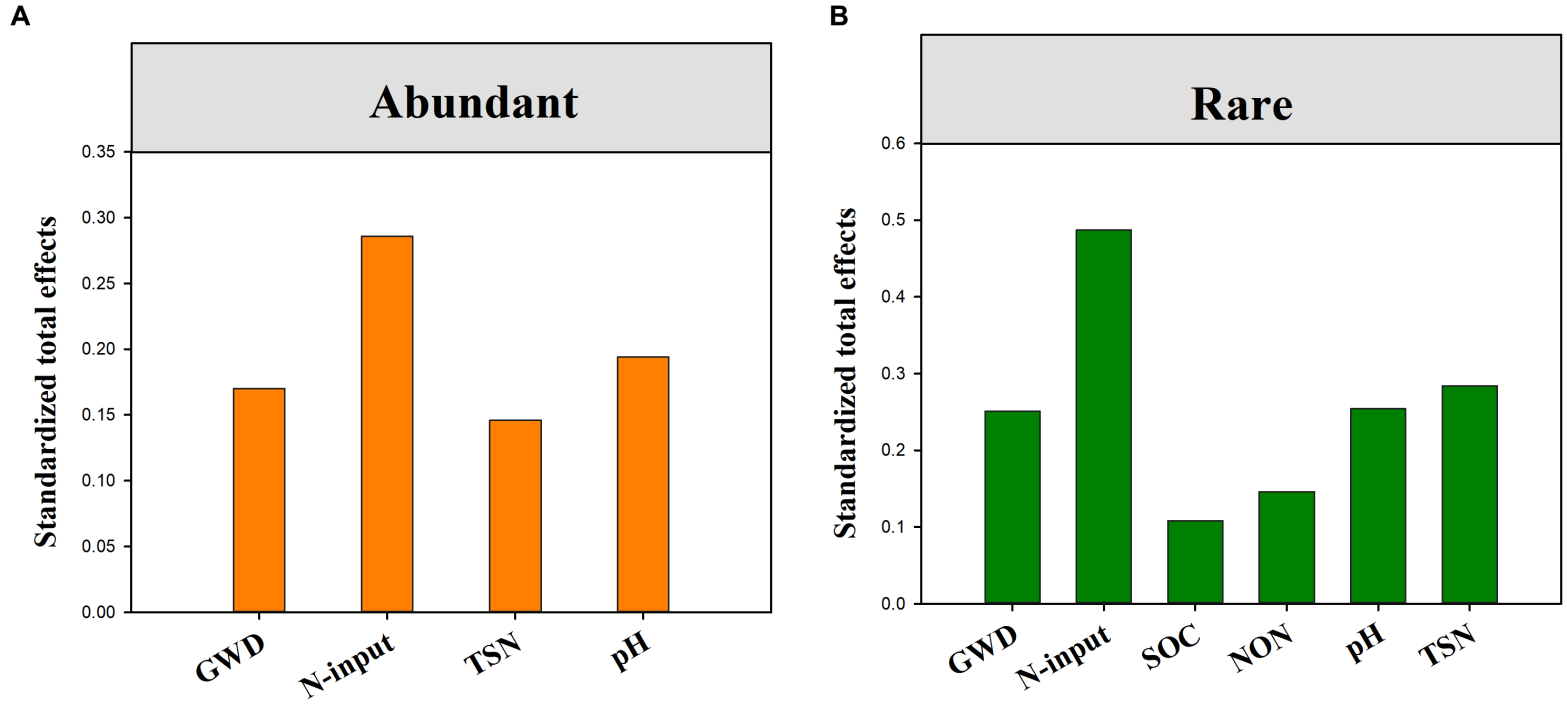
The standardized total effects (direct plus indirect effects) of groundwater depth, reduced nitrogen application and soil properties on abundant **(A)** and rare sub-communities **(B)** derived from the SEM model.

The fitted SEM showed that reduced nitrogen application, changes in groundwater depth and soil attributes collectively explained 15.80 and 45.50% of the variation in the community composition of abundant and rare bacteria, respectively (Figures 5A,B). Notably, groundwater depth could directly and indirectly affect the species composition of rare bacteria, but only had an indirect impact on that of abundant bacteria. Contrarily, reduced nitrogen application could directly and indirectly affect the species composition of both abundant and rare bacteria. Moreover, standardized total effects derived from the SEM revealed that the species composition of abundant bacteria was primarily determined by reduced nitrogen application, followed by soil pH, TSN and groundwater depth, while that of rare bacteria was primarily regulated by reduced nitrogen application, followed by TSN, groundwater depth, pH, NON, and SOC (Figures 6, 7).

## 4. Discussion

### 4.1. Different responses of abundant and rare bacterial composition to reduced nitrogen application and groundwater depth change

Global environmental change such as nitrogen overload has been recognized as one of the major threats to biodiversity and ecosystems. Nitrogen fertilization can significantly alter soil microbial community composition (She et al., 2018; Bai et al., 2020; Liu W. et al., 2021). Moreover, groundwater plays a key role in shaping soil microbial community composition (Zhang et al., 2020). Previous studies have also found that soil rare and abundant microbial taxa adopt different strategies to cope with environmental changes (Hou et al., 2020; Liang et al., 2020; Jiao and Lu, 2020b). However, how differently rare and abundant bacterial sub-communities respond to reduced nitrogen application, groundwater depth change and their interactions remains largely unclear. In this study, we observed that reduced nitrogen application led to a substantial change in both abundant and rare bacterial composition, which is similar to the findings of our study on denitrifier communities (Bai et al., 2020). However, groundwater depth and its interactions with reduced nitrogen application only significantly influenced the community composition of rare bacteria. SEM further demonstrated that reduced nitrogen application could directly and indirectly affect both abundant and rare sub-communities, while groundwater depth had no direct influence on abundant sub-communities. We also observed that reduced nitrogen application and groundwater depth change resulted in a greater shift in rare species composition than in abundant species composition, which is inconsistent with the findings of a previous report regarding soil microbial responses to climatic changes (Liang et al., 2020). This result implies that abundant bacterial composition is more stable to local environmental changes than rare bacterial composition in the agricultural ecosystem. From the above results, we suggest that the response of soil microbes to reduced nitrogen application and groundwater depth change varies between abundant and rare taxa.

Abundant and rare microbial sub-communities respond differently to the environment (Jiao and Lu, 2020b; Wang et al., 2021b). The difference in functional traits between abundant and rare species may mediate their response to environmental conditions by influencing their fitness and performance (McGill et al., 2006; Webb et al., 2010). Abundant species may have broader tolerances (Kurm et al., 2017; Delgado-Baquerizo et al., 2018), which enables them to perform well under different environmental conditions (Jiao and Lu, 2020b). On the contrary, rare species may have worse performance as they are more specialized in rare resources (Umaña et al., 2015). Hence, our study observed that abundant bacteria were distributed more ubiquitously and exhibited a wider habitat niche breadth than rare bacteria. Consequently, abundant species can occupy diverse niches and have greater tolerance and adaptability to environmental changes than rare species (Jiao and Lu, 2020b). Therefore, reduced nitrogen application and groundwater depth change may select species with higher resistance and resource competitiveness within the community, and filter out stress-avoidant rare species, which may account for why rare sub-communities were more sensitive to reduced nitrogen application and groundwater depth change than abundant sub-communities.

Taxa with small populations may be more susceptible to extinction under rapid global environmental changes (Storch et al., 2018). Rare species play key roles in regulating the health and function of ecosystems (Zhang Y. et al., 2019; Xiong et al., 2021). Given that rare sub-communities were more sensitive to reduced nitrogen application and groundwater depth change than abundant sub-communities, we hypothesized that over-nitrogen application, groundwater depth change and their interactions may greatly negatively influence the health and function of the agricultural ecosystem by altering the community composition of rare microbes. Collectively, we highlight that future cropland management should explore the appropriate nitrogen input and groundwater table levels to maintain the compositional stability of soil microbes, especially rare taxa.

### 4.2. Different influences of the variation in soil attributes on abundant and rare bacterial composition

Both reduced nitrogen application and groundwater depth change can remarkably alter soil attributes, thereby resulting in a shift in soil microbial composition (Bai et al., 2020). Thus, in this study the composition of abundant and rare sub-communities was altered by the variations in soil attributes caused by reduced nitrogen application and groundwater depth change (Supplementary Table S1), which is consistent with the previous studies on denitrifier communities (Bai et al., 2020). In addition, the response direction and strength to soil attributes may vary significantly between abundant and rare bacteria (Jiao and Lu, 2020b; Wang et al., 2021a). Similarly, both abundant and rare bacterial sub-community composition showed the same direction of responses to the changes in soil attributes, while rare bacteria had stronger response strength (i.e., Mantel *R*) than abundant bacteria. This result suggests that rare bacterial sub-communities may be more sensitive to the changes in soil attributes caused by reduced nitrogen application and groundwater depth change.

Soil microbial communities were controlled by a wide range of soil variables such as soil pH (Tripathi et al., 2018; Jiao and Lu, 2020b), soil salinity (Zhang K. et al., 2019), and soil fertility (Jiao and Lu, 2020a). Among soil attributes, the community composition of rare bacteria was primarily determined by soil TSN, followed by pH, NON, and SOC, while that of abundant bacteria was only shaped by soil pH and TSN, which may suggest that soil factors affect abundant and rare bacterial sub-communities differently. More importantly, both soil pH and TSN had the most important influence on the composition of abundant and rare bacteria, which indicates that reduced nitrogen application and groundwater depth change alter soil bacterial composition primarily by regulating soil pH and nutrients. Soil pH and nutrient availability can directly affect bacterial growth and interact with abiotic and biotic factors to regulate soil bacterial community structure (Delgado-Baquerizo et al., 2018). In addition, soil pH and nutrient availability determine soil microbial community structure in different ecosystem types (Fierer and Jackson, 2006). For example, soil pH and nutrient availability have the most powerful influence on abundant and rare sub-communities in large-scale agricultural ecosystems (Jiao and Lu, 2020a,b). However, our previous study has observed that soil nutrients and salinity play more important roles in shaping the composition of denitrifying communities (Bai et al., 2020). Here are several interpretations of the results. Firstly, the strong covariation among different variables made it difficult to precisely quantify their influence. Secondly, such discrepancies may be also partly due to the differences in the environmental regime between 1- and 2-year experiments and the different sensitivity of microbial taxa to soil attributes (Chase et al., 2017; Ji et al., 2020; Liang et al., 2020). Taken together, we emphasize the different influences of soil-factor variations on abundant and rare bacterial composition.

### 4.3. Research limitation and future perspectives

This study provided first-hand evidence that abundant and rare bacterial sub-communities had different responses to reduced nitrogen application, groundwater depth change and their interactions. Rare sub-communities were more sensitive to the changes in soil attributes caused by reduced nitrogen application and groundwater depth change. Notably, our study conducted a short-term experiment (2 years) and only selected two levels of N input, which may lead to a probable biased result. Hence, a longer observation period (>5 years) and more nitrogen input levels and microbial taxa should be considered in further research to better elucidate the intrinsic influence of nitrogen fertilization on the agricultural ecosystem, and the appropriate nitrogen input amount should be accurately determined to maintain the health and function of agricultural ecosystems.

## 5. Conclusion

This study conducted a comprehensive comparison of the response of abundant and rare bacterial sub-communities to reduced nitrogen application, groundwater depth change, their interactions and soil factors. The community composition of rare bacteria was significantly altered by reduced nitrogen application, groundwater depth change and their interactions in summer maize field, while that of abundant bacteria was regulated by reduced nitrogen application alone. Moreover, SEM demonstrated that reduced nitrogen application and groundwater depth change could also alter the species composition of abundant and rare bacteria by causing significant variations in summer maize soil attributes. The variations in soil pH and TSN caused by reduced nitrogen application and groundwater depth played the most important role in altering the community composition of abundant and rare bacteria, respectively. More importantly, rare bacterial sub-communities were more sensitive to these local environmental changes. Collectively, our findings provide first-hand evidence that abundant and rare microbial sub-communities of summer maize soil respond differently to reduced nitrogen application and groundwater depth change.

## Data availability statement

The data presented in the study are deposited in the NCBI repository, accession number PRJNA1021577.

## Author contributions

FB, XQ, and PL conceptualized the study. FB carried out field research and gathered information. FB, XQ, and ZD created the techniques. FB and WG wrote the manuscript. DQ was in charge of language editing. All authors read and approved the final manuscript.
