# Supplementary material for: Different responses of abundant and rare bacterial composition to groundwater depth and reduced nitrogen application in summer maize field
Source: Front Microbiol. 2023 Oct 13;14:1220731. doi: 10.3389/fmicb.2023.1220731 (PMC10613034; doi:10.3389/fmicb.2023.1220731)
Supplement: Supplementary file 1 [file Data_Sheet_1.pdf]

Table S1 Effects of depression of groundwater table, reduced nitrogen application and their interactions on soil attributes

	pH		EC		SOC		TSN		TSP		SAP		NON		NHN	
	F	*P*	*F*	*P*	*F*	*P*	*F*	*P*	*F*	*P*	*F*	*P*	*F*	*P*	*F*	*P*
Ninput	6.33	<0.05	250.54	<0.0001	11.915	<0.05	17.632	<0.001	10.803	<0.01	101.244	<0.0001	0.613	>0.05	10.472	<0.01
GWD	26.71	<0.0001	7.67	<0.05	0.638	>0.05	0.783	>0.05	4.411	<0.05	0.225	>0.05	11.108	<0.01	4.389	<0.05
Ninput*GWD	10.11	<0.01	38.7	<0.0001	0.476	>0.05	12.649	<0.01	1.361	>0.05	2.706	>0.05	0.449	>0.05	12.159	<0.01

Ninput, levels of N input; groundwater depth levels; GWD, groundwater depth levels; Ninput*GWD, interaction between Ninput and GWD; SOC, soil organic carbon; TSN, soil total nitrogen; TSP, soil total phosphor; SAP, soil available phosphorus;pH, soil pH; EC, soil electrical conductivity; NHN, soil NH_4^+-N contents, NON, soil $NO_3^{—}$-N content; F, F-test Value; *P*, probability value

Table S2 General description of all, abundant and rare OTUs data sets

Index	OTU numbers	Sequence numbers
**All OTUs**	4,619	746,976
**Abundant OTUs**	203(4.39%)	36,2101(50.52%)
**Rare OTUs**	3,096(67.03%)	86,975(12.13%)

OTUs with a relative abundance >0.1 % of the total abundance were defined as abundant bacteria, and rare bacteria were defined as those with a relative abundance <0.01% .

Table S3 Observed phyla and their relative abundance from rare and abundant taxa

Phylum	Rare taxa	Abundant taxa
Chloroflexota	14.66	11.18
Pseudomonadota	14.21	29.17
Actinobacteriota	10.48	25.46
Acidobacteriota	9.31	19.55
Bacteroidota	7.96	2.14
Myxococcota	7.32	
Gemmatimonadota	6.34	4.88
Planctomycetota	5.35	
Bacillota	5.19	3.35
Patescibacteria	4.36	
Verrucomicrobiota	3.68	
Bdellovibrionota	1.89	
Cyanobacteria	1.76	1.25
Elusimicrobiota	1.11	
Others	6.38	3.02

Compared with https://doi.org/10.1099/ijsem.0.005056, we have changed *Proteobacteria* to *Pseudomonadota*, changed *Chloroflexi* to *Chloroflexota*

PSEUDOMONADOTA CORRIG. PHYL. NOV. (current name)

Correction of the effectively published synonym: Proteobacteria (sic) Garrity et al. 2005.

CHLOROFLEXOTA CORRIG. PHYL. NOV.(current name)

Correction of the effectively published synonym: Chloroflexi (sic) Garrity and Holt 2001.

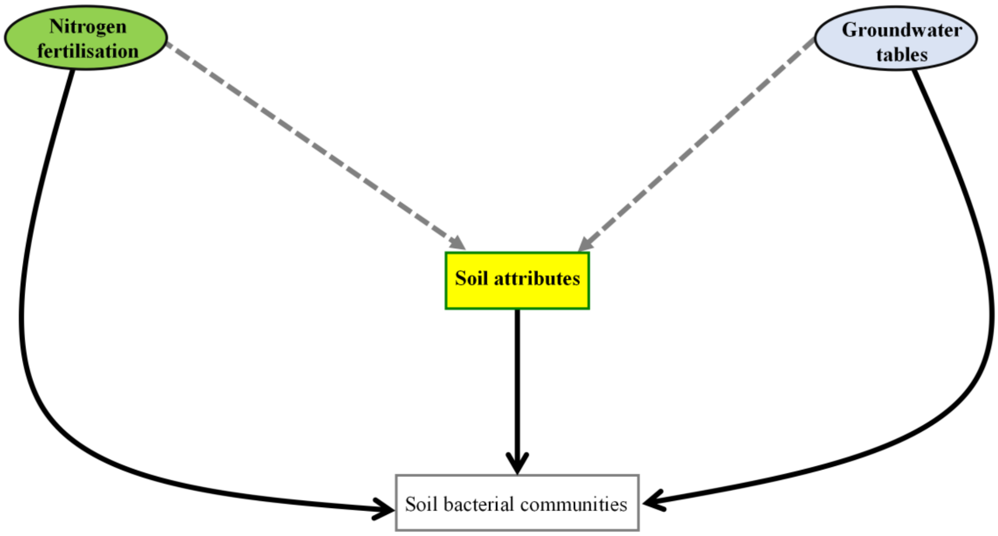

Figure S1 Priori structural equation models including direct and indirect effects of altered groundwater depth, reduced nitrogen application and soil attributes on the soil bacterial composition of abundant and rare taxa.
